# Comparison of Various Obesity-Related Indices for Identification of Metabolic Syndrome: A Population-Based Study from Taiwan Biobank

**DOI:** 10.3390/diagnostics10121081

**Published:** 2020-12-12

**Authors:** Tai-Hua Chiu, Ya-Chin Huang, Hsuan Chiu, Pei-Yu Wu, Hsin-Ying Clair Chiou, Jiun-Chi Huang, Szu-Chia Chen

**Affiliations:** 1 Department of General Medicine, Kaohsiung Medical University Hospital, Kaohsiung Medical University, Kaohsiung 807, Taiwan; tata14080222@gmail.com (T.-H.C.); mickey990055@gmail.com (H.C.); 2 Department of Preventive Medicine, Kaohsiung Municipal Ta-Tung Hospital, Kaohsiung Medical University, Kaohsiung 801, Taiwan; jasimine0603@gmail.com; 3 Department of Occupational & Environmental Medicine, Kaohsiung Medical University Hospital, Kaohsiung Medical University, Kaohsiung 807, Taiwan; 4 Department of Internal Medicine, Kaohsiung Municipal Siaogang Hospital, Kaohsiung Medical University, Kaohsiung 812, Taiwan; wpuw17@gmail.com (P.-Y.W.); scarchenone@yahoo.com.tw (S.-C.C.); 5 Division of Nephrology, Department of Internal Medicine, Kaohsiung Medical University Hospital, Kaohsiung Medical University, Kaohsiung 807, Taiwan; 6 Teaching and Research Center of Kaohsiung Municipal Siaogang Hospital, Kaohsiung Medical University, Kaohsiung 812, Taiwan; phoenixchiou@gmail.com; 7 Faculty of Medicine, College of Medicine, Kaohsiung Medical University, Kaohsiung 807, Taiwan; 8 Research Center for Environmental Medicine, Kaohsiung Medical University, Kaohsiung 807, Taiwan

**Keywords:** obesity, triglyceride–glucose index, visceral adiposity index, metabolic syndrome, population-based study, biobank

## Abstract

This study aimed to evaluate the performance of 11 obesity-related indices, including body mass index (BMI), waist circumference, waist-to-height ratio, waist–hip ratio, a body shape index, abdominal volume index, body adiposity index, body roundness index, conicity index, visceral adiposity index (VAI), and triglyceride glucose (TyG) index, in identifying metabolic syndrome (MetS) in adults. The information of 5000 participants was obtained from the Taiwan Biobank. Logistic regression analyses were performed to determine the associations between MetS and obesity-related indices with odds ratio (ORs). The predictive performance of the indices to identify MetS was compared using receiver operating characteristic (ROC) curves and areas under curves (AUCs). Multivariate-adjusted logistic regression showed that the ORs for MetS increased across the quartiles of each index. ROC curves analysis demonstrated that TyG index had the greatest AUC in men (AUC = 0.850) and women (AUC = 0.890). Furthermore, VAI had the greatest AUC in men (AUC = 0.867) and women (AUC = 0.925) aged 30−50 years, while TyG index had the greatest AUC in men (AUC = 0.849) and women (AUC = 0.854) aged 51−70 years. Among the studied obesity-related indices, TyG index and VAI exhibited the best performance for identifying MetS in adults. TyG index and VAI may be the relevant indices to assess MetS in clinical practice.

## 1. Introduction

Metabolic syndrome (MetS) is a cluster of risk factors for cardiovascular (CV) diseases and diabetes mellitus (DM), including abdominal obesity, hyperglycemia, elevated blood pressure and dyslipidemia [1]. MetS is associated with a high risk of developing DM [2], chronic kidney disease [3], stroke [4], CV diseases and all-cause mortality [5,6]. The prevalence of MetS ranges from 11.9% to 49.0% in the Asia-Pacific region, depending on age, ethnicity and race of the population [7]. In Taiwan, the prevalence of MetS greatly increased from 13.6% to 25.5% based on two official Nutrition and Health Surveys conducted 12 years apart [8]. Considering the growing prevalence of MetS and its impact on public health, early identification and management of MetS is important to prevent the subsequent development of type 2 DM, CV diseases and other complications. In clinical screening, it would be helpful to have a simple index to identify individuals at high risk of MetS.

The pathogenesis of MetS is complex and still not well understood, although visceral adiposity has been demonstrated to play a major role in most of the pathogenic pathways involved in MetS [9,10]. Numerous studies have discussed the predictive ability of obesity and lipid-related indices in identifying metabolic abnormalities. Body mass index (BMI) is a simple measurement of obesity status, while waist circumference (WC) reflects abdominal adiposity and may represent visceral adiposity better than BMI [11]. Waist–hip ratio (WHR) and waist-to-height ratio (WHtR) are anthropometric indices based on WC and also reflect abdominal fat, and have been reported to be better indicators of MetS than BMI [12,13]. Abdominal volume index (AVI) is used to assess general volume, and it has been highly associated with dysfunction of glucose metabolism [14]. In addition, body roundness index (BRI), an indicator of body adiposity [15], has been shown to have better predictive value for MetS than BMI and WHR [16]. Other indices, such as conicity index (CI), a body shape index (ABSI) and body adiposity index (BAI) have also been used in epidemiological studies that explored their relationships with metabolic disorders [16,17,18]. In addition to traditional indices, several parameters that combine anthropometric and biochemical measurements have recently been proposed. Among these emerging indices, visceral adiposity index (VAI) is sex-specific and has been reported to be a better indicator of MetS than other traditional indices [19]. Triglyceride glucose (TyG) index, based on triglycerides and fasting glucose, is another novel parameter that has been reported to be able to reflect insulin resistance [20] and identify metabolically unhealthy individuals [21].

Numerous studies have explored the associations between these indices and metabolic risk in different ethnicities and regions. However, in Taiwan, few studies have investigated the utility of obesity-related parameters in identifying MetS in the general population. Thus, this study aimed to evaluate the performance of 11 obesity-related indices in identifying MetS in Taiwanese adults.

## 2. Materials and Methods

### 2.1. Data Source and Study Population

The data used in the present study were collected from the Taiwan Biobank (TWB), a general population-based research database comprised of cancer-free residents aged 30−70 years enrolled through 31 recruitment stations in Taiwan since 2008. The data source of the TWB has previously been described [22,23]. The methodologies for data collection from all participants in TWB were the same and in a standardized procedure. Details on the TWB can be found on its official website (https://taiwanview.twbiobank.org.tw/index). Written informed consent was obtained from all enrolled participants and all investigations in this study were conducted in accordance with the Declaration of Helsinki. This study was approved by the Institutional Review Board of Kaohsiung Medical University Hospital (KMUHIRB-E(I)-20180242), approved on 03/08/2018. 

**Figure 1:**
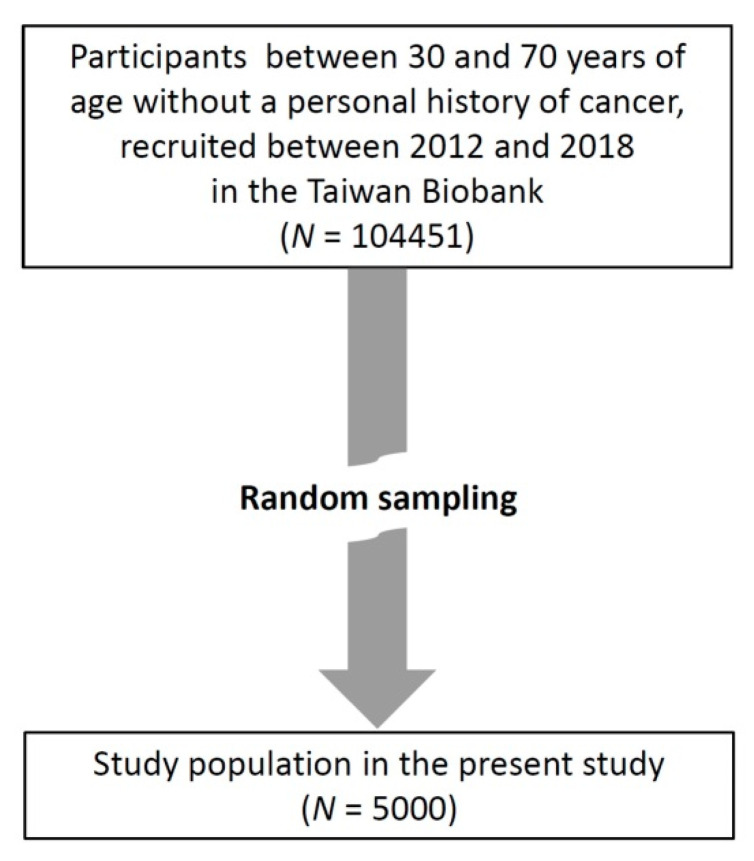
Flowchart of study population.

In the present study, we included a total of 5000 participants (2335 men and 2665 women) by random sampling from the 104451 participants recruited between 2012 and 2018 in TWB (Figure 1). The baseline demographic information and lifestyle patterns (current smoking and exercise habits) were obtained through a face-to-face interview with TWB researchers. Anthropometric measurements (BMI, WC, hip circumference [HC]), systolic (SBP) and diastolic blood pressure (DBP), as well as overnight fasting blood chemistry parameters (fasting plasma glucose, high-density lipoprotein cholesterol [HDL-C], low-density lipoprotein cholesterol [LDL-C], triglycerides [TG], total cholesterol, uric acid, serum creatinine, and glycated hemoglobin [HbA1C]) of all participants were collected. Pulse pressure was defined as the difference between SBP and DBP. Estimated glomerular filtration rate (eGFR) was calculated using the Chronic Kidney Disease Epidemiology Collaboration (CKD-EPI) formula [24].

### 2.2. Definition of MetS

The diagnosis of MetS in the present study was based on the definition of MetS from the Administration of Health Promotion in Taiwan, which is derived from the National Cholesterol Education program Adult Treatment Panel-III [25]. Participants with any three of the following risk factors were considered to have MetS: (1) central obesity (WC ≥90 cm for men, ≥80 cm for women); (2) high blood pressure (SBP ≥130 mmHg and/or DBP ≥85mmHg); (3) low HDL-C (HDL-C <40 mg/dL for men, <50 mg/dL for women); (4) increased fasting plasma glucose (≥100 mg/dL); and (5) elevated TG level (≥150 mg/dL).

### 2.3. Calculations of Obesity-Related Indices

The obesity-related indices, including BMI, WHtR, WHR, ABSI, AVI, BAI, BRI, CI, VAI and TyG index were calculated using the following equations [14,15,20,26,27,28,29]:

BMI = weight (kg)/height^2^ (m)
WHtR = WC (cm)/height (cm)
WHR = WC (cm)/HC (cm)
ABSI = WC (m)/[BMI^2/3^(kg/m^2^) × height^1/2^(m)]
AVI = [2 × WC^2^(cm) + 0.7×(WC−HC)^2^(cm)]/1000
BAI = [HC (m)/height^2/3^(m)]−18
BRI = 364.2−365.5[1−π^−2^WC^2^(m)Height^−2^(m)]^1/2^
CI = 0.109^−1^WC (m)[Weight (kg)/Height (m)]^−1/2^
VAI male = [WC (cm)/39.68−1.88 × BMI (kg/m^2^)] × [TG (mmol/L)/1.03] × [1.31/HDL (mmol/L)]
VAI female = [WC (cm)/36.58−1.89 × BMI (kg/m^2^)] × [TG (mmol/L)/0.81] × [1.52/HDL (mmol/L)]
TyG index = Ln [fasting TG (mg/dL) × fasting plasma glucose (mg/dL)/2]

### 2.4. Statistical Analysis

The study participants were stratified into MetS and non-MetS groups. The data were presented as percentages for categorical variables, and mean ± standard deviation for continuous variables. Categorical variables were compared using the Chi-square test, and continuous variables were compared using the independent *t*-test. Logistic regression analyses were performed to determine the associations between MetS and the 11 obesity-related indices with odds ratios (ORs) and 95% confidence intervals before and after adjusting for confounding factors including age, sex, pulse pressure, total cholesterol, LDL-C, uric acid, current smoking status, eGFR, and exercise habits. The first quartile was used as a reference to calculate the ORs for MetS of the 2nd, 3rd, and 4th quartiles of each obesity-related index. Receiver operating characteristic (ROC) curves were generated to assess the performance of the different obesity-related indices in identifying MetS. Areas under the curves (AUCs) were calculated to compare the predictive value of the various indices to identify MetS. Delong et al.’s nonparametric approach was used to compare the AUCs of each index [30]. Statistical analyses were performed using SPSS version 22.0 for Windows (IBM Corp, Armonk, NY, USA). Comparisons between AUCs and calculations of the Youden index were performed using MedCalc for Windows, version 15.0 (MedCalc Software, Ostend, Belgium).

## 3. Results

### 3.1. Characteristics of the Study Population

Comparisons of the clinical characteristics between the MetS and non-MetS groups are shown in Table 1. Of the 5000 participants, 2335 (46.7%) were men. Overall, 22.1% of the men and 17.9% of the women had MetS. The men with MetS were more likely to be older, have higher SBP and DBP, higher levels of uric acid, HbA1C, fasting glucose and TG, higher values of obesity-related indices, a higher prevalence of current smoking, and a lower level of eGFR compared to those without MetS. The differences in the clinical characteristics between the women with and without MetS were similar to those in the men. In addition, the women with MetS were more likely to have higher levels of total cholesterol and LDL-C compared to the women without MetS.

### 3.2. ORs for MetS Risk Across Quartiles of Each Obesity-Related Index

We divided each obesity-related parameter into quartiles and used unadjusted and multivariate-adjusted logistic regression analyses to assess the associations with MetS across quartiles of these parameters. After adjustments for age, pulse pressure, total cholesterol, LDL-C, eGFR, uric acid, smoking status and exercise habits, all obesity-related indices were significantly associated with MetS in both men and women, except for WC, ABSI, AVI, and BAI in women. The ORs for MetS increased across the quartiles of each obesity-related index (Table 2).

### 3.3. ROC Curve Analysis for the Obesity-Related Indices in Identifying MetS

Table 3 and Table 4 demonstrate the ROC analysis and AUCs of the 11 obesity-related indices in identifying MetS in men and women, respectively. Among the obesity-related indices, TyG index had the greatest AUC both in men (AUC = 0.850, cutoff value = 8.83) and women (AUC = 0.890, cutoff value = 8.70). In men, VAI had the second-highest diagnostic ability for MetS (AUC = 0.845, cutoff value = 1.74), followed by WC (AUC = 0.828, cutoff value = 89.8) and AVI (AUC = 0.828, cutoff value = 16.2). In women, VAI (AUC = 0.880, cutoff value = 1.83) and WC (AUC = 0.820, cutoff value = 80.1) had the second and third greatest AUCs, respectively, while VAI showed no difference with TyG index for predicting MetS (*p* > 0.05 compared with TyG index). TyG index had the highest Youden index values in both men and women.

**Figure 2:**
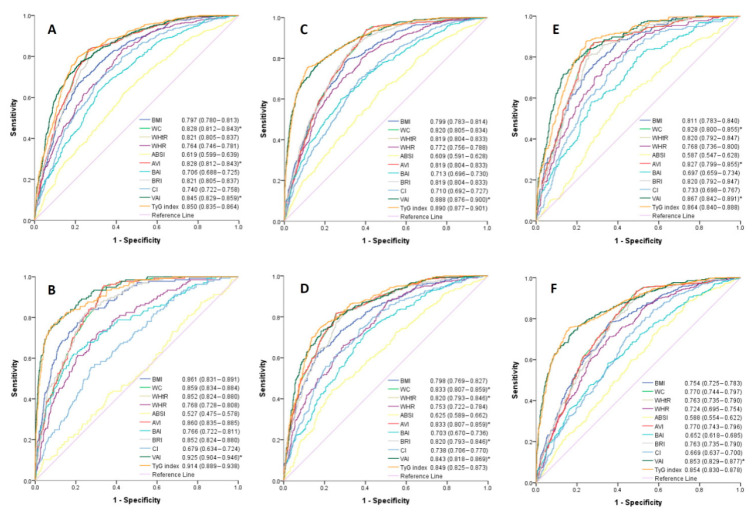
Comparison of the predictive value of 11 obesity-related parameters for diagnosis of metabolic syndrome among (**A**) all males, (**B**) all females, (**C**) males aged 30−50 years, (**D**) females aged 30−50 years, (**E**) males aged 51−70 years, and (**F**) females aged 51−70 years. * *p*-value > 0.05 when compared with area under curve of TyG index using the DeLong method.

Figure 2 shows the ROC curves and AUCs of the 11 obesity-related indices for MetS in men and women stratified by two decades of age (30−50 and 51−70 years). VAI had the greatest AUC in men (AUC = 0.867) and women (AUC = 0.925) aged 30−50 years, while TyG index had the greatest AUC in both men and women aged 51−70 years (AUC = 0.849 in men; 0.854 in women). However, the differences between the AUCs of TyG index and VAI were still not significant in these two age groups in both sexes.

## 4. Discussion

The present study evaluated the predictive ability and cutoff value of 11 obesity-related parameters, including BMI, WC, WHR, WHtR, AVI, CI, ABSI, BRI, BAI, VAI, and TyG index, in identifying MetS among adults enrolled in the TWB project. Overall, we found that TyG index and VAI were practical parameters in identifying MetS in both men and women. In addition, TyG index and VAI had the highest predictive performance in identifying MetS in different age groups (30−50 years and 51−70 years) in both sexes.

MetS consists of CV risk factors such as hypertension and other metabolic risk factors, and the common mechanisms of these abnormalities have been suggested to be related to visceral adiposity [9]. Visceral adipose tissue (VAT) has been reported to play a more important role in insulin resistance than subcutaneous adipose tissue, because it is related to higher inflammatory cytokine production [31]. Dysregulation of adipokines, which is also related to abnormal visceral fat accumulation, may result in dyslipidemia and hypertension [9]. VAI was introduced in 2010 by Amato et al. and was found to be linked to CV risk [29]. A recent systematic review indicated that VAI was a practical predictor for type 2 DM in Asian populations [32]. Moreover, in a study of 10.000 Iranian individuals aged from 35 to 65 years, Baveicy et al. found that VAI had a better predictive value for MetS than BRI and ABSI [19]. There are several possible reasons to explain why VAI outperforms the other obesity-related indices in predicting MetS. First, VAI is highly correlated with WC, TG, and HDL, three major components of MetS criteria. Second, VAI has been shown to be inversely associated with adiponectin, a protective adipocytokine, and associated with high levels of inflammation-related cytokines [33]. This may further contribute to insulin resistance and CV abnormalities. Third, although glucose metabolism is not included in its formula, VAI has been reported to be positively associated with insulin resistance assessed using the homeostatic model assessment (HOMA-IR) [34] and also to be a predictor of DM [32].

TyG index is another lipid-related metabolic parameter that consists of fasting plasma glucose and TG, and is well known to be a robust marker of insulin resistance [20]. Lee et al. demonstrated that TyG index was significantly associated with the risk of developing DM, and that it had better performance than triglyceride/HDL–cholesterol ratio and HOMA-IR in people without diabetes [35]. In addition, Yu et al. found that TyG index could be used to identify components of MetS and individuals at high risk of cardiometabolic diseases [21]. Several possible mechanisms may explain the impact of TyG index on the development of MetS. TyG index has been associated with incident hypertension in a Chinese population [36], possibly due to insulin resistance-related hyperinsulinemia and a subsequent increase in sympathetic nervous system activity [37] and renin-angiotensin-aldosterone system activation [38]. Furthermore, HDL level has been shown to be inversely associated with insulin resistance [39]. This implies that insulin resistance may be the link between TyG index and dyslipidemia, which is also a significant component of MetS. Furthermore, TyG index exhibited the second highest AUC to identify low HDL-C and had a comparable AUC to identify high blood pressure among these 11 obesity-related indices (Table S1). This finding indicates that TyG index has its important role for identifying 4 of 5 MetS components, but has weakness in identifying central obesity.

In the present study, VAI and TyG index exhibited greater AUCs in identifying MetS than other traditional obesity-related parameters. The cutoff values of VAI to predict MetS were 1.74 in men and 1.83 in women in our study, compared to 4.1 and 4.2 in men and women, respectively, in an Iranian population [19], and 2.0 in middle-aged and elderly Chinese populations [40]. The difference between our study and the work by Baveicy et al. [19] may be due to dietary and ethnic differences, as our results were closer to those of the reported Chinese populations [40]. In addition, different criteria used to diagnose MetS in previous studies may also have influenced the cutoff values. The TyG index cutoff values to predict MetS in our study (8.8 in men and 8.7 in women) were very close to the findings in other studies of Asian populations [40,41]. The predictive value of TyG index for MetS has been reported to be inferior to VAI in previous studies, possibly because the measurement of TyG index does not include WC, a crucial marker of central obesity [40]. However, in the present study, TyG index and VAI showed comparable ability in identifying MetS. Given that TyG index is highly associated with insulin resistance, which is considered to be the consequence of excess VAT accumulation [42], this may suggest that TyG index can also reflect visceral adiposity.

Some sex differences were noted in our study. In both age groups of women (30−50 years and 51−70 years), TyG index and VAI showed approximately the same AUCs in identifying MetS (*p* > 0.05), and these AUCs were significantly greater than those of the other 9 obesity-related parameters. In men, TyG index had the largest AUC compared to the other parameters; however, the AUCs of TyG index and VAI were similar to those of AVI and WC (*p* > 0.05). In addition, in men aged 51−70 years, TyG index and VAI had significantly larger AUCs than ABSI, BAI, CI, BMI, and WHR, but there were no significant differences compared with AVI, WC, BRI, and WHtR. TyG index and VAI seemed to perform better in women than in men in identifying MetS, as they outperformed all other 9 indices in women; however, their AUCs for MetS were similar to those of some other parameters in men. The reasons for these discrepancies between men and women and the results of previous studies remain to be investigated, although it may be associated with sex differences in the distribution of adiposity deposition. Although women have higher body fat percentage than men, women tend to have more subcutaneous adipose tissue, and men tend to have more VAT [43]. The crucial form of fat that results in insulin resistance and subsequent metabolic derangement is VAT [42]. This may suggest that the underlying mechanisms of MetS and the impact of VAT are different between men and women. In addition, WC has been reported to increase with aging even in the absence of weight gain [44]. This may partially explain the different results between men in the two age groups (30−50 years and 51−70 years), as WC is a major factor in the formulae of traditional anthropometric obesity-related parameters.

ABSI was introduced in 2012 based on the National Health and Nutrition Examination Survey (NHANES) 1999–2004 data, and it has been related to VAT and premature mortality [26]. However, previous studies have reported conflicting results regarding its utility in identifying metabolic risk factors. Fujita et al. reported that ABSI did not show a better ability than BMI or WC for predicting DM, hypertension, and dyslipidemia in Japanese adults [45], whereas Bawadi et al. found that ABSI was better than BMI in predicting the risk of DM in a Qatari population [18]. Our results showed that ABSI was the weakest predictor for MetS regardless of age or sex among all 11 obesity-related indices. This finding is in agreement with a previous study [45] that indicated that ABSI was not practical in identifying MetS. Ethnic differences may help to explain the relatively poor predictive power of ABSI, which was mainly developed using data from Western populations [26]. Moreover, Asian populations have been reported to have a greater amount of abdominal adipose tissue compared with Western populations [46]. The relatively lower average height of Asian populations may also alter the implications of ABSI in identifying MetS.

There are several limitations to this study. First, the cross-sectional design cannot evaluate longitudinal relationships between these obesity-related parameters and MetS. It was primarily relevance descriptions of data. Further prospective studies are needed to explore possible causal associations. Second, alcohol intake, sleeping, and economic status were not included in our analysis. These factors may also be associated with the development of MetS. Third, the participants in our study were limited to Taiwan; therefore, our results might not be generalizable to other populations.

## 5. Conclusions

In conclusion, the present study indicated that both TyG index and VAI are the most valuable indices among the obesity-related indices to identify MetS in Taiwanese adults. TyG index and VAI can be easily calculated through routine laboratory examinations and simple anthropometric measurements, and therefore can be used as relevant assessment tools for MetS in clinical practice.

## Figures and Tables

**Table 1:** Clinical characteristics of the study participants classified by the presence of MetS.

	Men (*n* = 2335)			Female (*n* = 2665)		
Characteristics	MetS (−)	MetS (+)	*p*-Value	MetS (−)	MetS (+)	*p*-Value
*n* (%)	1819 (77.9)	516 (22.1)		2187 (82.1)	478 (17.9)	
Age (year)	49.0 ± 11.1	51.8 ± 10.5	<0.001	48.4 ± 10.2	54.8 ± 9.3	<0.001
Systolic BP (mmHg)	116.9 ± 14.7	128.0 ± 16.6	<0.001	108.8 ± 15.6	127.1 ± 17.7	<0.001
Diastolic BP (mmHg)	73.9 ± 10.0	80.8 ± 11.0	<0.001	66.4 ± 9.5	74.9 ± 11.0	<0.001
Current smoking (%)	17.4	25.6	<0.001	1.1	2.3	0.047
Exercise habits (%)	46.9	45.3	0.535	41.9	47.7	0.010
**Laboratory parameters**						
Uric acid (mg/dL)	6.3 ± 1.3	6.9 ± 1.5	<0.001	4.7 ± 1.0	5.6 ± 1.3	<0.001
HbA1C (%)	5.7 ± 0.7	6.3 ± 1.2	<0.001	5.6 ± 0.5	6.4 ± 1.2	<0.001
Fasting glucose (mg/dL)	96.5 ± 17.4	112.0 ± 30.1	<0.001	90.5 ± 10.8	108.9 ± 29.1	<0.001
Total cholesterol (mg/dL)	192.3 ± 34.3	195.5 ± 38.5	0.082	194.9 ± 35.7	205.0 ± 39.6	<0.001
TG (mg/dL)	114.0 ± 68.5	212.7 ± 151.2	<0.001	87.3 ± 47.1	179.0 ± 119.3	<0.001
HDL-C (mg/dL)	51.3 ± 11.1	41.3 ± 8.5	<0.001	61.3 ± 12.9	47.4 ± 9.2	<0.001
LDL-C (mg/dL)	123.9 ± 31.6	121.6 ± 34.2	0.162	119.4 ± 31.7	127.9 ± 35.1	<0.001
eGFR (mL/min/1.73 m^2^)	97.5 ± 14.7	94.8 ± 16.1	<0.001	106.6 ± 12.8	100.2 ± 14.5	<0.001
**Obesity-related indices**						
BMI (kg/m^2^)	24.4 ± 2.9	27.8 ± 3.3	<0.001	22.8 ± 3.1	26.5 ± 3.5	<0.001
WC (cm)	85.7 ± 7.6	95.3 ± 7.6	<0.001	79.1 ± 8.3	89.3 ± 8.5	<0.001
WHtR	0.5077 ± 0.0455	0.5640 ± 0.0441	<0.001	0.5036 ± 0.0561	0.5727 ± 0.0576	<0.001
WHR	0.8870 ± 0.0510	0.9364 ± 0.0479	<0.001	0.8371 ± 0.0631	0.9003 ± 0.0592	<0.001
ABSI	0.0786 ± 0.0038	0.0802 ± 0.0037	<0.001	0.0787 ± 0.0052	0.0807 ± 0.0054	<0.001
AVI	14.9 ± 2.6	18.3 ± 3.0	<0.001	12.8 ± 2.6	16.2 ± 3.2	<0.001
BAI	26.1 ± 2.8	28.3 ± 3.1	<0.001	30.0 ± 3.5	33.0 ± 4.2	<0.001
BRI	3.54 ± 0.88	4.68 ± 0.96	<0.001	3.49 ± 1.10	4.89 ± 1.32	<0.001
CI	1.23 ± 0.06	1.28 ± 0.56	<0.001	1.21 ± 0.08	1.28 ± 0.08	<0.001
VAI	1.35 ± 1.01	3.21 ± 2.87	<0.001	1.26 ± 0.96	3.44 ± 3.12	<0.001
TyG index	8.48 ± 0.50	9.20 ± 0.54	<0.001	8.19 ± 0.44	9.02 ± 0.56	<0.001
**Components of MetS**						
Central obesity (%)	26.3	83.3	<0.001	43.3	95.6	<0.001
High BP (%)	25.2	68.8	<0.001	11.8	60.0	<0.001
Low HDL-C (%)	9.7	54.7	<0.001	16.1	71.8	<0.001
High TG (%)	17.8	69.8	<0.001	5.3	57.5	<0.001
Dysglycemia (%)	20.1	69.8	<0.001	8.8	59.0	<0.001

Abbreviations: MetS, metabolic syndrome; BP, blood pressure; HbA1C, glycated hemoglobin; TG, triglycerides; HDL-C, high-density lipoprotein cholesterol; LDL-C, low-density lipoprotein cholesterol; eGFR, estimated glomerular filtration rate; BMI, body mass index; WC, waist circumference; WHtR, waist-to-height ratio; WHR, waist–hip ratio; ABSI, a body shape index; AVI, abdominal volume index; BAI, body adiposity index; BRI, body roundness index; CI, conicity index; VAI, visceral adiposity index; TyG index, triglyceride glucose index.

**Table 2:** Unadjusted and multivariate-adjusted odds ratios (ORs) for MetS stratified by quartiles of each obesity-related parameter.

	All		Men		Women	
	Unadjusted OR	Adjusted OR	Unadjusted OR	Adjusted OR	Unadjusted OR	Adjusted OR
BMI						
Q1	1	1	1	1	1	1
Q2	3.757 (2.544−5.546)	3.271 (2.190−4.885)	6.461 (3.543−11.782)	6.233 (3.375−11.511)	4.140 (2.322−7.383)	3.208 (1.766−5.826)
Q3	9.614 (6.662−13.874)	8.211 (5.606−12.028)	13.438 (7.508−24.052)	13.789 (7.595−25.034)	9.813 (5.673−16.974)	6.034 (3.416−10.656)
Q4	30.539 (21.339−43.707)	28.172 (19.295−41.134)	43.547 (24.548−77.249)	49.268 (27.242−89.103)	31.872 (18.677−54.389)	18.232 (10.438−31.849)
WC						
Q1	1	1	1	1	1	1
Q2	7.205 (4.264−12.175)	6.982 (4.100−11.891)	6.060 (2.953−12.437)	5.693 (2.749−11.792)	0.962 (0.406−2.282) *	0.763 (0.317−1.841) *
Q3	15.262 (9.119−25.542)	14.156 (8.346−24.013)	17.041 (8.587−33.817)	16.246 (8.094−32.606)	17.559 (9.167−33.634)	12.948 (6.640−25.246)
Q4	59.523 (35.914−98.653)	61.112 (36.123−103.390)	65.446 (33.233−128.881)	66.175 (33.122−132.210)	40.734 (21.428−77.437)	21.584 (11.105−41.949)
WHtR						
Q1	1	1	1	1	1	1
Q2	6.561 (3.844−11.197)	5.358 (3.118−9.207)	3.806 (2.080−6.964)	3.661 (1.982−6.760)	4.657 (2.148−10.096)	3.476 (1.582−7.638)
Q3	24.319 (14.604−40.499)	18.637 (11.106−31.275)	14.015 (7.991−24.579)	13.385 (7.550−23.729)	24.541 (11.945−50.421)	15.773 (7.574−32.844)
Q4	67.034 (40.434−111.133)	47.220 (28.172−79.148)	43.680 (25.087−76.055)	42.257 (23.899−74.718)	58.995 (28.891−120.466)	28.357 (13.588−59.176)
WHR						
Q1	1	1	1	1	1	1
Q2	2.341 (1.703−3.219)	2.096 (1.507−2.914)	3.865 (2.371−6.301)	3.774 (2.296−6.204)	3.203 (1.908−5.378)	2.665 (1.563−4.543)
Q3	6.103 (4.556−8.176)	5.221 (3.819−7.136)	9.789 (6.162−15.552)	8.681 (5.400−13.958)	7.924 (4.882−12.860)	5.325 (3.219−8.809)
Q4	14.155 (10.647−18.817)	11.474 (8.385−15.701)	19.825 (12.561−31.289)	18.116 (11.307−29.027)	21.453 (13.393−34.363)	11.420 (6.944−18.782)
ABSI						
Q1	1	1	1	1	1	1
Q2	1.602 (1.275−2.013)	1.375 (1.080−1.751)	1.691 (1.230−2.327)	1.676 (1.205−2.333)	1.568 (1.133−2.172)	1.192 (0.836−1.700) *
Q3	2.300 (1.849−2.861)	1.796 (1.421−2.271)	2.297 (1.686−3.129)	2.074 (1.498−2.872)	2.162 (1.581−2.957)	1.672 (1.186−2.356)
Q4	3.014 (2.434−3.731)	2.135 (1.688−2.702)	3.246 (2.402−4.387)	2.772 (2.000−3.843)	2.924 (2.158−3.963)	1.591 (1.125−2.249)
AVI						
Q1	1	1	1	1	1	1
Q2	7.658 (4.702−12.471)	7.220 (4.400−11.849)	5.453 (2.829−10.509)	5.082 (2.611−9.893)	1.822 (0.835−3.978) *	1.446 (0.651−3.208) *
Q3	16.626 (10.355−26.693)	15.323 (9.423−24.917)	16.510 (8.829−30.873)	15.882 (8.394−30.052)	21.692 (11.339−41.498)	16.350 (8.417−31.762)
Q4	57.011 (35.757−90.899)	56.690 (34.853−92.208)	56.437 (30.409−104.743)	57.456 (30.498−108.241)	48.899 (25.703−93.026)	26.343 (13.567−51.150)
BAI						
Q1	1	1	1	1	1	1
Q2	2.239 (1.762−2.847)	2.611 (2.019−3.376)	2.197 (1.512−3.193)	2.231 (1.514−3.286)	1.730 (1.180−2.538)	1.265 (0.840−1.905) *
Q3	2.437 (1.921−3.091)	3.329 (2.548−4.350)	4.286 (3.011−6.099)	3.958 (2.740−5.718)	3.089 (2.161−4.417)	1.787 (1.216−2.627)
Q4	4.776 (3.810−5.988)	7.167 (5.404−9.506)	7.153 (5.069−10.092)	6.397 (4.455−9.186)	7.205 (5.132−10.115)	3.482 (2.404−5.045)
BRI						
Q1	1	1	1	1	1	1
Q2	6.478 (3.794−11.059)	5.286 (3.075−9.087)	3.481 (1.929−6.281)	3.329 (1.828−6.065)	4.627 (2.134−10.032)	3.456 (1.573−7.593)
Q3	24.336 (14.614−40.524)	18.646 (11.112−31.290)	13.141 (7.617−22.669)	12.493 (7.162−21.793)	24.797 (12.070−50.942)	15.922 (7.647−33.152)
Q4	66.926 (40.369−110.954)	47.134 (28.120−79.004)	40.480 (23.644−69.306)	39.027 (22.439−67.878)	59.089 (28.935−120.666)	28.257 (13.541−58.965)
CI						
Q1	1	1	1	1	1	1
Q2	2.105 (1.578−2.807)	1.683 (1.248−2.268)	2.927 (1.915−4.474)	2.755 (1.786−4.249)	2.706 (1.790−4.091)	1.958 (1.266−3.027)
Q3	4.955 (3.799−6.461)	3.806 (2.882−5.027)	5.766 (3.855−8.625)	5.444 (3.595−8.244)	5.303 (3.589−7.835)	3.663 (2.424−5.533)
Q4	9.111 (7.034−11.803)	5.940 (4.509−7.826)	13.122 (8.865−19.422)	11.649 (7.718−17.582)	8.606 (5.875−12.607)	4.152 (2.746−6.276)
VAI						
Q1	1	1	1	1	1	1
Q2	5.698 (3.322−9.774)	5.265 (3.034−9.136)	5.406 (2.800−10.440)	5.533 (2.832−10.810)	9.720 (3.445−27.427)	7.010 (2.438−20.156)
Q3	14.342 (8.559−24.032)	12.501 (7.354−21.251)	12.449 (6.624−23.396)	13.547 (7.092−25.875)	23.562 (8.586−64.657)	15.629 (5.572−43.835)
Q4	96.894 (58.439−160.652)	89.441 (53.092−150.676)	71.236 (38.366−132.268)	87.431 (45.876−166.629)	187.779 (69.450−507.713)	130.431 (47.352−359.274)
TyG index						
Q1	1	1	1	1	1	1
Q2	6.641 (3.679−11.986)	5.355 (2.939−9.757)	5.169 (2.589−10.321)	5.410 (2.681−10.917)	7.112 (2.764−18.299)	4.991 (1.909−13.047)
Q3	16.292 (9.226−28.768)	13.213 (7.391−23.621)	15.564 (8.079−29.983)	16.726 (8.571−32.640)	16.497 (6.625−41.083)	9.952 (3.913−25.310)
Q4	128.485 (73.550−224.451)	152.608 (85.593−272.093)	76.152 (39.903−145.331)	104.850 (53.364−206.009)	160.309 (65.637−391.533)	101.466 (40.691−235.015)

* *p*-value > 0.05. Values expressed as odds ratio (OR) and 95% confidence interval. Covariates in the multivariable-adjusted model included age, pulse pressure, total cholesterol, LDL-C, eGFR, uric acid, smoking status and exercise habits.

**Table 3:** Area under curve (AUC), cutoff value, Youden index, sensitivity and specificity of 11 obesity-related indices for predicting MetS in men.

	AUC (95% Confidence Interval)	Cutoff Value	Sensitivity (%)	Specificity (%)	Youden Index
BMI	0.797 (0.780−0.813)	26.0	69.8	75.7	0.454
WC	0.828 (0.812−0.843) *	89.8	83.3	73.7	0.571
WHtR	0.821 (0.805−0.837)	0.53	81.2	71.6	0.528
WHR	0.764 (0.746−0.781)	0.894	83.9	56.4	0.403
ABSI	0.619 (0.599−0.639)	0.079	62.0	56.5	0.185
AVI	0.828 (0.812−0.843) *	16.2	83.3	73.7	0.570
BAI	0.706 (0.688−0.725)	27.2	63.4	68.4	0.318
BRI	0.821 (0.805−0.837)	3.94	81.2	71.6	0.528
CI	0.740 (0.722−0.758)	1.25	72.1	65.2	0.372
VAI	0.845 (0.829−0.859) *	1.74	77.7	77.2	0.549
TyG index	0.850 (0.835−0.864)	8.83	79.7	78.9	0.585

All *p*-value < 0.05, except for * *p*-value > 0.05 when compared with AUC of TyG index using the DeLong method.

**Table 4:** Area under curve (AUC), cutoff value, Youden index, sensitivity and specificity of 11 obesity-related indices for predicting MetS in women.

	AUC (95% Confidence Interval)	Cutoff Value	Sensitivity (%)	Specificity (%)	Youden Index
BMI	0.799 (0.783−0.814)	23.9	78.4	69.8	0.482
WC	0.820 (0.805−0.834)	80.1	93.3	60.0	0.533
WHtR	0.819 (0.804−0.833)	0.521	85.8	66.4	0.522
WHR	0.772 (0.756−0.788)	0.852	79.7	62.2	0.419
ABSI	0.609 (0.591−0.628)	0.079	63.8	53.4	0.172
AVI	0.819 (0.804−0.833)	13.0	94.1	59.6	0.537
BAI	0.713 (0.696−0.730)	31.7	60.0	72.2	0.322
BRI	0.819 (0.804−0.833)	3.77	85.8	66.4	0.522
CI	0.710 (0.692−0.727)	1.23	69.4	63.5	0.329
VAI	0.888 (0.876−0.900) *	1.83	77.6	83.2	0.608
TyG index	0.890 (0.877−0.901)	8.70	75.5	88.6	0.641

All *p*-value < 0.05, except for * *p*-value > 0.05 when compared with AUC of TyG index using the DeLong method.

## Supplementary Materials

The following are available online at https://www.mdpi.com/2075-4418/10/12/1081/s1, Table S1: Comparison of AUCs for obesity-related indices in MetS components in all study subjects.
