# Supplementary material for: Comparison of Various Obesity-Related Indices for Identification of Metabolic Syndrome: A Population-Based Study from Taiwan Biobank
Source: Diagnostics (Basel). 2020 Dec 12;10(12):1081. doi: 10.3390/diagnostics10121081 (PMC7763700; doi:10.3390/diagnostics10121081)
Supplement: Supplementary file 1 [file diagnostics-10-01081-s001.pdf]

Table S1. Comparison of AUCs for obesity-related indices in MetS components in all study subjects

	Central Obesity		High Blood Pressure		Low HDL-C		Elevated TG		Increased Fasting Glucose	
Component	**AUC**	***p***	**AUC**	***p***	**AUC**	***p***	**AUC**	***p***	**AUC**	***p***
BMI	0.847(0.837-0.858)	<0.001	0.672(0.656-0.688)	<0.001	0.651(0.633-0.669)	<0.001	0.705(0.688-0.721)	<0.001	0.656(0.639-0.674)	<0.001
WC	0.903(0.895-0.910)	<0.001	0.681(0.665-0.697)	<0.001	0.631(0.613-0.649)	<0.001	0.710(0.694-0.727)	<0.001	0.673(0.656-0.690)	<0.001
WHtR	0.949(0.944-0.954)	<0.001	0.681(0.665-0.697)	<0.001	0.651(0.634-0.669)	<0.001	0.683(0.666-0.700)	<0.001	0.661(0.644-0.678)	<0.001
WHR	0.804(0.793-0.816)	<0.001	0.675(0.659-0.691)	<0.001	0.599(0.581-0.618)	<0.001	0.691(0.674-0.708)	<0.001	0.674(0.657-0.691)	<0.001
ABSI	0.724(0.710-0.738)	<0.001	0.571(0.554-0.589)	<0.001	0.523(0.504-0.542)	0.019	0.539(0.520-0.558)	<0.001	0.569(0.551-0.588)	<0.001
AVI	0.904(0.896-0.912)	<0.001	0.680(0.664-0.696)	<0.001	0.632(0.614-0.650)	<0.001	0.709(0.693-0.726)	<0.001	0.672(0.655-0.688)	<0.001
BAI	0.782(0.770-0.795)	<0.001	0.561(0.543-0.579)	<0.001	0.614(0.595-0.632)	<0.001	0.536(0.517-0.556)	<0.001	0.532(0.513-0.551)	<0.001
BRI	0.949(0.944-0.954)	<0.001	0.681(0.665-0.697)	<0.001	0.651(0.634-0.669)	<0.001	0.683(0.666-0.700)	<0.001	0.661(0.644-0.678)	<0.001
CI	0.853(0.842-0.863)	<0.001	0.632(0.615-0.649)	<0.001	0.581(0.563-0.600)	<0.001	0.618(0.600-0.636)	<0.001	0.625(0.607-0.643)	<0.001
VAI	0.714(0.700-0.728)	<0.001	0.607(0.590-0.624)	<0.001	0.867(0.856-0.877)	<0.001	0.971(0.967-0.976)	<0.001	0.638(0.620-0.656)	<0.001
TyG index	0.664(0.649-0.679)	<0.001	0.645(0.629-0.662)	<0.001	0.734(0.718-0.750)	<0.001	0.986(0.983-0.989)	<0.001	0.746(0.731-0.761)	<0.001
